# Supplementary material for: The Development of a Prediction Model Based on Random Survival Forest for the Postoperative Prognosis of Pancreatic Cancer: A SEER-Based Study
Source: Cancers (Basel). 2022 Sep 25;14(19):4667. doi: 10.3390/cancers14194667 (PMC9563591; doi:10.3390/cancers14194667)
Supplement: Supplementary file 1 [file cancers-14-04667-s001.zip › cancers-1904450-supplementary.pdf]

Supplementary Material

The Deepsur model

The Deepsur model was developed by the pycox module, which was a derivative of pytorch. The different hyperparameters, including layers, nodes, learning rate, optimizer, had the influence on the eventual performance of the model. In our study, we optimize our model using the grid search strategy.The final structure of the Deepsur model were shown in Figure S1. As was shown in Figure S2, the best learning rate was 0.475. And the Adam algorithm was applied as the optimizer.

The optimal cut-off values of RSF risk stratification

The optimal cut-off values of RSF risk stratification were based on the X-tile software. The X-tile was a bio-informatics tool for biomarker assessment and outcome-based cut-point optimization. We used X-tile to select the best cut-off values for high-risk, medium-risk, and low-risk populations in the training set. The cut-off values were summarized in Table S2. To improve the clinical implement, we taken a rounded approach to the optimal cut-off values. Finally, the bets cut-off values for the above-mentioned populations were 123, 157.

Table S1 The results of the multivariable Cox regression analysis

Characteristics	HR	Lower.95	Uper.95	*P* value
Age	1.02	1.01	1.02	<0.001
Radiation				
No		reference		
Yes	1.08	0.98	1.2	0.126
Chemotherapy				
No		reference		
Yes	0.7	0.63	0.79	<0.001
Histological type				
Epithelial neoplasms		reference		
Adenomas and adenocarcinomas	1.38	0.92	2.06	0.116
Cystic, mucinous and serous	0.93	0.59	1.48	0.771
Ductal and lobular neoplasms	1.68	1.12	2.53	0.012
Complex epithelial neoplasms	1.5	0.89	2.54	0.128
Surg				
Local excision		reference		
Partial pancreatectomy	2.37	0.58	9.61	0.227

Local or partial pancreatectomy and duodenectomy	2.49	0.62	9.99	0.199
Total pancreatectomy	3.04	0.74	12.53	0.125
Total pancreatectomy and subtotal gastrectomy or duodenectomy	2.61	0.65	10.58	0.178
Extended pancreatoduodenectomy	2.45	0.6	9.94	0.212
Pancreatectomy	2.12	0.47	9.47	0.326
AJCC stage				
I		reference		
II	1.57	1.23	2.01	<0.001
III	1.22	0.59	2.54	0.589
IV	2.49	1.85	3.34	<0.001
T stage				
T1		reference		
T2	1.14	0.89	1.47	0.303
T3	1.33	1.03	1.72	0.031
T4	2.52	1.25	5.09	0.01

N stage				
N0		reference		
N1	1.15	1.01	1.31	0.034
Site				
PancreasHead		reference		
PancreasBodyTail	0.78	0.66	0.92	0.004
other	0.94	0.8	1.12	0.5
Clinical grade				
I		reference		
II	2.28	1.93	2.69	<0.001
III	3.1	2.62	3.68	<0.001
IV	3.69	2.56	5.32	<0.001
Tumor size	1	1	1.01	<0.001
Positive lymph nodes	1.01	1	1.03	0.077
Positive lymph nodes rate (%)	1.98	1.47	2.66	<0.001

Table S2 The best cut-off values of the RSF risk stratification with X-tile

Number	% Total	Events	Rate	Rank	Range
1343	47.90	654	48.70	0 to 1342	2.12 thru 123.46
742	26.46	696	93.80	1343 to 2084	123.48 thru 156.53
719	25.64	699	97.22	2085 to 2803	156.64 thru 211.96
2804	100.00	2049	73.07	0 to 2803	2.12 thru 211.96

Figure S1 The structure of the Deepsur model

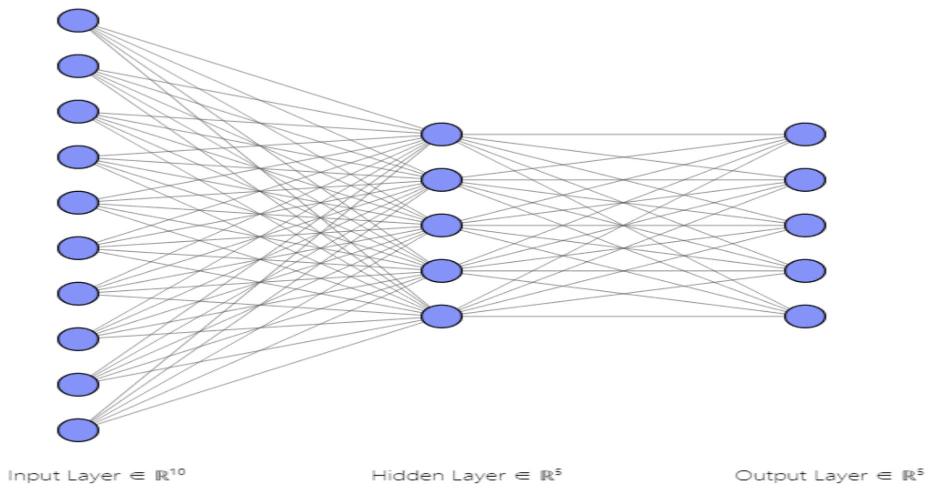

Figure S2 The learning rate plot

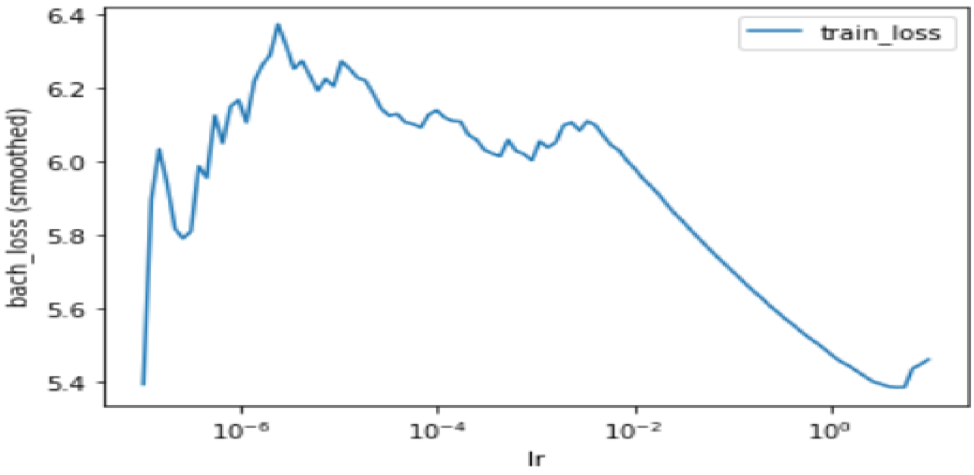

Figure S3 The survival analysis of risk factors in the RSF model

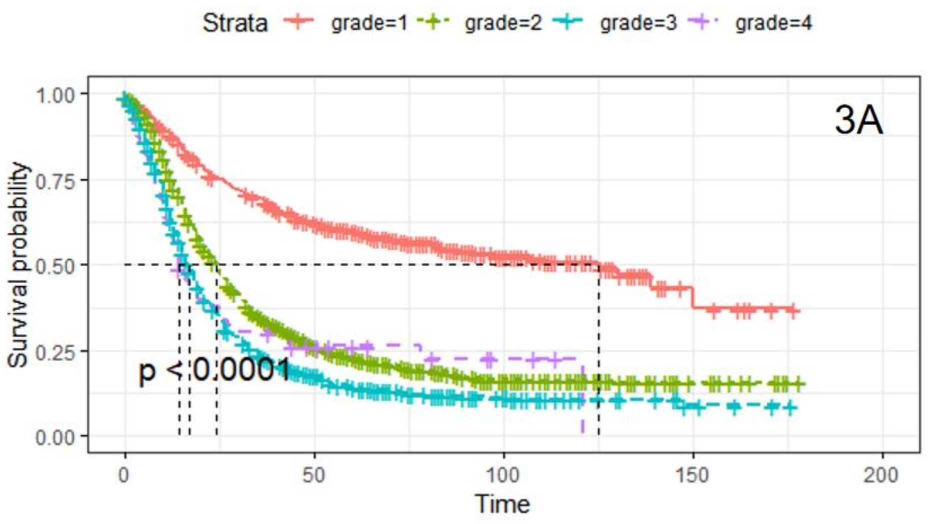

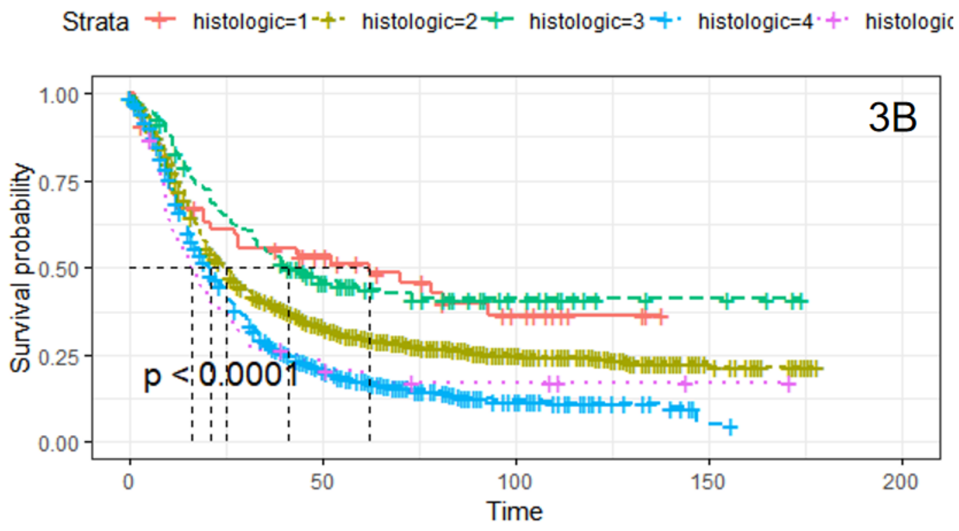

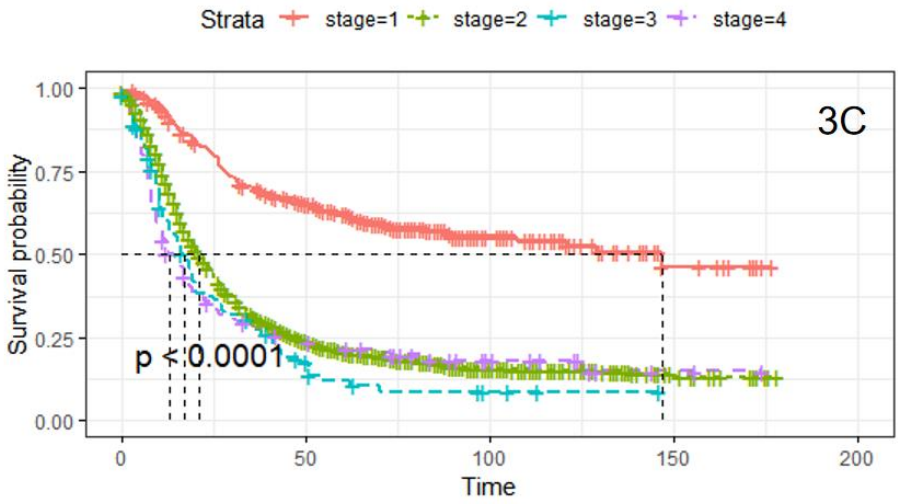
Strata stage=1 stage=2 stage=3 stage=4
3C
Survival probability
1.00
0.75
0.50
0.25
0.00
p < 0.0001
0
50
100
150
200
Time

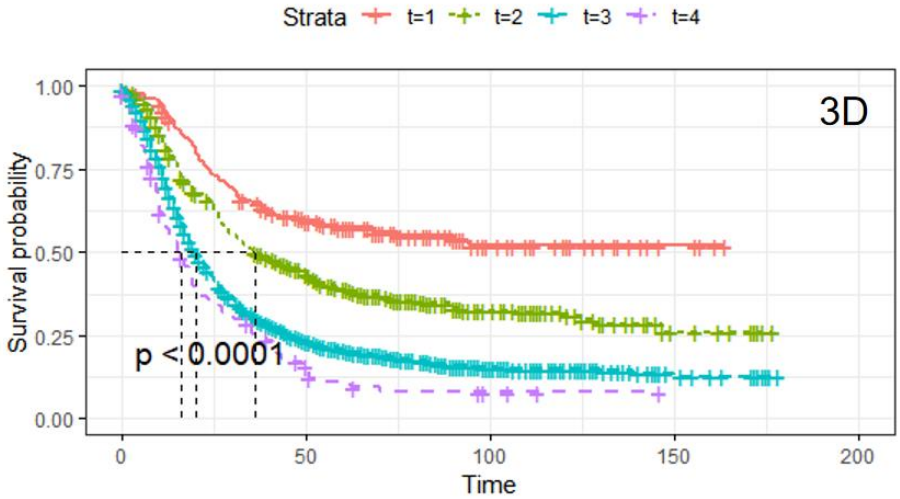
Strata t=1 t=2 t=3 t=4
3D
Survival probability
1.00
0.75
0.50
0.25
0.00
p < 0.0001
0
50
100
150
200
Time

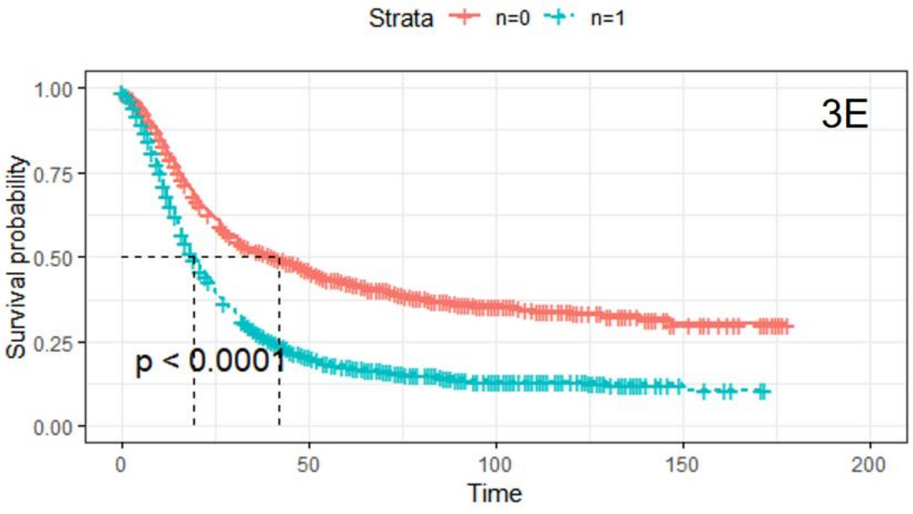
Strata n=0 n=1
3E
Survival probability
1.00
0.75
0.50
0.25
0.00
p < 0.0001
0
50
100
150
200
Time

Note: (3A): The survival analysis of the clinical grade. (3B): The survival analysis of histologic type. Histologic type 1: epithelial neoplasms; Histologic type 2: adenomas and adenocarcinomas; Histologic 3: cystic, mucinous, and serous neoplasms; Histologic type 4: ductal and lobular neoplasms; Histologic type 5: complex epithelial neoplasms. (3C): The survival analysis of AJCC stage. (3D): The survival analysis of T stage. (3E): The survival analysis of N stage.
